# Sociodemographic and Clinical Characteristics of Transgender Adults in Australia

**DOI:** 10.1089/trgh.2018.0019

**Published:** 2018-12-26

**Authors:** Ada S. Cheung, Olivia Ooi, Shalem Leemaqz, Pauline Cundill, Nicholas Silberstein, Ingrid Bretherton, Emily Thrower, Peter Locke, Mathis Grossmann, Jeffrey D. Zajac

**Affiliations:** ^1^ Department of Medicine (Austin Health), The University of Melbourne, Heidelberg, Australia.; ^2^ Adelaide Medical School, The University of Adelaide, Adelaide, Australia.; ^3^ Equinox Clinic, Thorne Harbour Health, Fitzroy, Australia.

**Keywords:** attention deficit disorder with hyperactivity, autistic disorder, depression, gender dysphoria, transgender persons

## Abstract

***Background:*** Over the last 10 years, increases in demand for transgender health care has occurred worldwide. There are few data on clinical characteristics of Australian adult transgender individuals. Understanding gender identity patterns, sociodemographic characteristics, gender-affirming treatments, as well as medical and psychiatric morbidities, including neurobehavioral conditions affecting transgender and gender-diverse adults will help to inform optimal health service provision.

***Purpose:*** In an Australian adult transgender cohort, we aimed to first, assess referral numbers and describe the sociodemographic and clinical characteristics, and second, to specifically assess the prevalence of autism spectrum disorder (ASD) and attention-deficit/hyperactivity disorder (ADHD).

***Methods:*** We performed a retrospective audit of deidentified electronic medical records in a primary care and a secondary care gender clinic in Melbourne, Australia. Annual referral rates, sociodemographic data, and prevalence of medical and psychiatric conditions were obtained.

***Results:*** Data for 540 transgender individuals were available. Rapid rises were observed in referrals for transgender health services, more than 10 times the number in 2016 compared with 2011. Median age at initial presentation was 27 years (interquartile range (22, 36), range 16–74). Around 21.3% were unemployed and 23.8% had experienced homelessness despite high levels of education. Around 44.1% identified as trans male, 36.3% as trans female, and 18.3% as gender nonbinary. Medical morbidities were rare but mental illness was very common. The prevalence of depression was 55.7%, anxiety in 40.4%, ADHD in 4.3%, and ASD in 4.8%, all higher than reported age-matched general Australian population prevalence.

***Conclusions:*** Rising demand for transgender care, socioeconomic disadvantage, and high burden of mental health conditions warrants a comprehensive multidisciplinary approach to provide optimal care for transgender individuals. Given that ASD and ADHD are prevalent, in addition to gender-affirming treatments, psychosocial interventions may assist individuals in navigating health care needs and to support social aspects of gender transition. Further studies are required to understand links between ASD, ADHD, and gender identity and to evaluate optimal models of health service provision for transgender individuals.

## Introduction

There is increasing recognition of the need for adequate health services to meet the needs of transgender individuals. Transgender people, whose gender identity is markedly and persistently incongruent with their biological sex, almost always experience gender dysphoria. Characterized by severe distress and discomfort, gender dysphoria compels transgender individuals to seek treatment. In addition to gender transition, complexities such as high rates of depression and potentially neurobehavioral conditions, such as autism spectrum disorder (ASD) or attention-deficit/hyperactivity disorder (ADHD) may require specific treatment and affect transition-related care.^1^ Addressing the specific health needs of individuals with gender dysphoria are required to optimize quality of life and social functioning.

Despite reported increases in demand for adult and pediatric transgender health services in recent years,^2–4^ there are few data on clinical characteristics of Australian adult transgender individuals. Gender-affirming interventions, including hormonal and surgical interventions are not well profiled.^5^ Increased understanding of gender identity patterns, sociodemographic and clinical characteristics of transgender, and gender diverse adults, including psychiatric and medical burden will help to inform health service provision.

Mental health conditions have been reported to have higher prevalence within transgender populations and there have been many reports of high rates of depression and anxiety^6–8^ occurring in transgender adults. There also has been a suggestion that personality disorders^9,10^ and eating disorders^11^ may be more prevalent among transgender individuals. Co-occurrence with ASD has been described in children and adolescents,^12,13^ and while much of the literature has associated autism traits detected on screening tests with gender dysphoria,^14,15^ only one study has assessed prevalence of the diagnosis of ASD among adult transgender individuals.^16^ However, not all studies have reported a higher prevalence of ASD in transgender individuals,^17,18^ suggesting that high scores on autism screening tests may be potentially related to high social anxiety. A potential higher prevalence of ADHD among 54 transgender individuals in an online survey has also recently been reported.^19^ Further study is warranted given that neurobehavioral conditions may affect assessment of gender dysphoria, require specific treatment, and affect the delivery of individualized supportive care.

Hormonal therapy for gender transition involves the administration of testosterone for masculinization, or estradiol for feminization. As testosterone and estradiol play important roles in cardiovascular disease,^20^ there is also uncertainty regarding cardiovascular risk among transgender individuals.^21^ Increased cardiovascular morbidity and mortality has been observed in retrospective clinical studies.^22,23^ This is plausible given that hormonal therapy with testosterone and estradiol both appear to be associated with lipid derangements and potentially worsening cardiovascular risk factors such as hypertension and insulin resistance.^24,25^ Further study is required.

We hypothesized first that there would be a rising number of transgender individuals seeking adult health services in Australia. Second, we hypothesized that gender identity distribution and demographic data would be different in endocrine specialist clinics compared with primary care. Specifically, as current Australian Pharmaceutical Benefits Scheme subsidy for testosterone therapy requires specialist assessment, we hypothesized that greater proportion of transgender males seeking testosterone therapy would be seen in the specialist setting. Third, given recent reports,^16,19^ we hypothesized that ADHD and ASD would be more prevalent among transgender adults attending gender clinics than those in the general population.

The aims of this descriptive study in adult transgender individuals were first, to document the number of new transgender individuals seeking health care; second, to describe the gender identity patterns, sociodemographic and clinical characteristics, including gender-affirming treatments of transgender individuals in Australia; and third, we aimed to specifically assess the prevalence of ADHD and ASD among this cohort of adult transgender individuals.

## Methods

A retrospective audit of electronic medical records was performed of new consultations for gender dysphoria at a primary care general practice clinic and an endocrine specialist clinic in Melbourne, Victoria, Australia. Individuals with gender dysphoria attending endocrine specialist clinics were compared those attending a primary care gender clinic, Equinox Gender Diverse Health Center operated by Thorne Harbour Health to obtain a more representative sample of transgender individuals in the community. In Australia, primary care general practitioners are the first point of medical care and play a central role in delivery of health care. A referral is required from a general practitioner to see an endocrine specialist. New consecutive consultations between 1st January 2011 and 31st December 2016 were analyzed. As the primary care clinic only commenced on 22nd February 2016, data were analyzed for the first 12 months of operation until 22nd February 2017. The study was approved by the Austin Health Human Research Ethics Committee (LNR/17/Austin/102) and the nature of the study did not require informed consent.

New presentations to both clinics (at initial consultation) were recorded in a deidentified manner. Self-reported gender identity as recorded on registration forms were classified into four groups: trans female (birth-assigned males who identified as female), trans male (birth-assigned females who identified as male), nonbinary (those that identified as neither male nor female), and unassigned (where individuals were undecided regarding their identity).

Sociodemographic parameters included age at presentation, residential postcode (classified using the Australian Standard Geographical Classification Remoteness Area (ASGC-RA) score), educational level, employment status, smoking status (nonsmoker, current smoker), hazardous alcohol intake (defined as >2 standard drinks per day on a regular basis or binge drinking), and history of homelessness was defined as those without a permanent residential address based on the Australian Bureau of Statistics official definition (lack of an adequate dwelling, lack of tenure or if living arrangements did not allow control of, or access to space for social relations).

Clinical characteristics recorded included medical morbidities and psychiatric conditions listed as diagnoses in the medical record by clinicians. Charlson Medical Comorbidity Index was calculated as a quantitative measure of overall medical morbidity.^26^ The Charlson Medical Comorbidity Index predicts mortality in individuals with multiple morbidities, such as heart disease, AIDS, or diabetes (a total of 22 conditions contribute to the score and low scores reflect low morbidity). A history of gender-affirming surgery and the gender-affirming hormone therapy regimens used were also recorded. To provide an additional assessment of cardiovascular risk, systolic and diastolic blood pressure (mmHg), height (cm), weight (kg), and body mass index (kg/m^2^, BMI) at initial consultation were also collected.

### Statistical analysis

Clinical characteristics are reported as median and interquartile range [median (IQR)] or number and proportion [*n* (%)] where appropriate. Statistical tests were performed as an exploratory analysis. Differences in sociodemographic and clinical characteristics between the two clinics were examined using Fisher's exact test and Student's *t*-test, or Mann–Whitney U test as its nonparametric alternative, as appropriate. A proportions test by *p*-value was performed to investigate differences in percentage of psychiatric conditions between the two clinics. The number of new consultations per year from the endocrine specialist clinic during 2011 to 2016 was also reported with a test for nonlinear trend. Statistical analysis was performed using R statistical software version 3.4.0. *p*<0.05 was considered statistically significant.

## Results

**FIG. 1.**
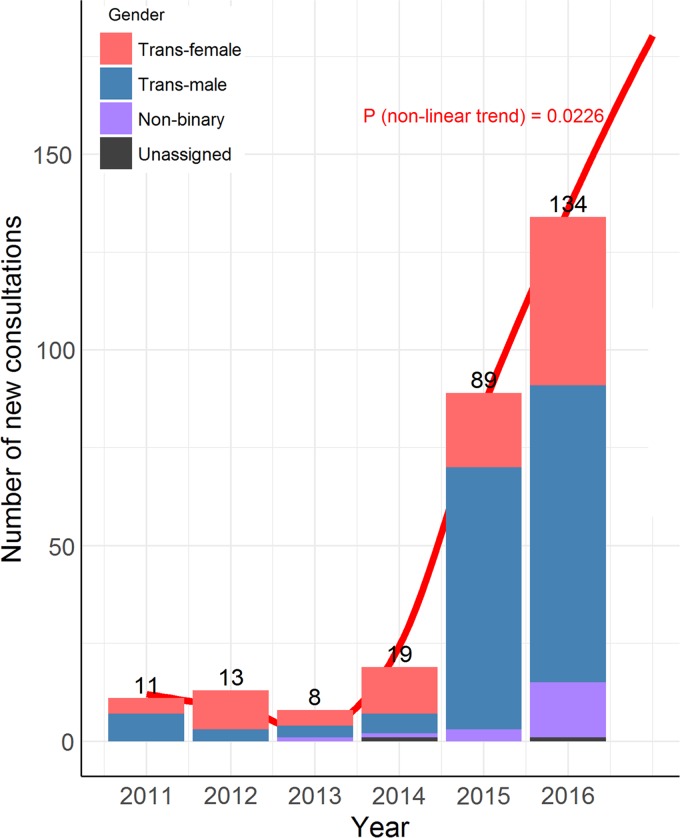
New consultations at endocrine specialist clinic. Number indicates the total number of new consultations during each year from January to December. Global test for nonlinear trend from inclusion of a second-degree polynomial term in Poisson regression (df=1; *F*-value=18.83; *p*=0.022).

Data were collected on a total of 540 new consultations; 283 from an adult endocrine specialist clinic and 257 from a primary care clinic. We observed a 10-fold increase in the number of transgender individuals newly attending endocrine specialist clinics between 2011 and 2016 (Fig. 1–Global test for nonlinear trend from inclusion of a second-degree polynomial term in Poisson regression (df=1; *F*-value=18.83; *p*=0.022)). Annualized data were not available for primary care.

### Sociodemographic characteristics

#### Gender identity

The gender identities of transgender individuals attending both practices are listed in Table 1. Significantly greater proportions of trans male individuals were seen in the endocrine specialist clinic compared with primary care (*p*<0.001 following pairwise tests with Bonferroni correction). Individuals identifying as gender nonbinary in primary care comprised 30.4% of all transgender individuals, and 7.4% in specialist clinics.

**Table 1.** Gender Identity Distribution Among Specialist and Primary Care Clinics

	All *N*=540	Endocrine specialist *N*=283	Primary care *N*=257	Overall *p* value
Trans female	196 (36.3%)	95 (33.6%)	101 (39.3%)	
Trans male	238 (44.1%)	165 (58.3%)	73 (28.4%)	
Nonbinary	99 (18.3%)	21 (7.4%)	78 (30.4%)	
Unassigned	7 (1.3%)	2 (0.7%)	5 (1.9%)	<0.001

Number (proportion of the cohort) is reported. *p* Value refers to overall difference in gender identity proportions based on Fisher's exact test.

#### Age and location of residence

Median age was 27 years (22, 36) with range 16–72 years. Telehealth consultations were frequently performed in endocrine specialist clinics with 31% of the 283 referred individuals residing in regional or remote areas of Australia (ASGC 1–5). Homelessness had been experienced by 23.8% of the total cohort.

#### Education and employment

Level of education was higher than age-matched Australian population mean with a formal nonschool qualification above secondary level attained in 73.5% of our cohort and 53.4% holding a university degree or higher (vs. 38.5% of the Australian population 25–29 years of age; two-proportions z-test chi-squared=27.57, df=1, *p*<0.001).^27^ Despite relatively high levels of education, overall unemployment rate was 21.3%, approximately four-fold higher than the Australian general population rate of 5.4%.^28^

#### Smoking and alcohol

Thirty-six percent were current smokers; three-fold higher than age-matched Australian population mean.^29^ Hazardous alcohol use in specialist clinics was greater than in primary care (15.8% vs. 8.0%, Chi-square=22.6; df=2; < 0.001); however, remained lower than the general Australian population.^29^

### Clinical characteristics

#### Medical morbidities

Median Charlson Medical Comorbidity Score was 0 (0, 1). Individual medical characteristics are described in Table 2. Median overall blood pressure was within normal limits (125/80 mmHg); however, median BMI was in the overweight range 25.6 kg/m^2^ (22.1, 30.9).

**Table 2.** Medical Characteristics

	All	*N*
Age at first consultation (years)	27 (22, 36)	540
Duration of hormone therapy (months)	0 (0, 18)	457
Charlson medical comorbidity index	0 (0, 1)	540
Hypertension^a^	46 (11.5%)	540
Hypercholesterolemia^b^	56 (15.6%)	540
Ischemic heart disease	4 (0.7%)	540
Human immunodeficiency virus (HIV)	2 (0.4%)	540
Chronic obstructive airways disease	79 (14.6%)	540
Liver disease	25 (4.6%)	540
Venous thromboembolism	7 (1.5%)	540
Stroke	1 (0.2%)	540
Cancer/Malignancy	8 (1.5%)	540
Previous genital reassignment surgery	46 (8.5%)	540
Body mass index (kg/m^2^)	25.6 (22.1, 30.9)	190
Systolic blood pressure (mmHg)	125 (120, 130)	397
Diastolic blood pressure (mmHg)	80 (75, 82)	394

Median (IQR) are shown or number (prevalence %) for categorical parameters. *N*=number of individuals for which data was available.

^a^ A hypertension diagnosis was based on antihypertensive treatment.

^b^ Hypercholesterolemia diagnosis was based on statin use.

#### Psychiatric conditions

**FIG. 2.**
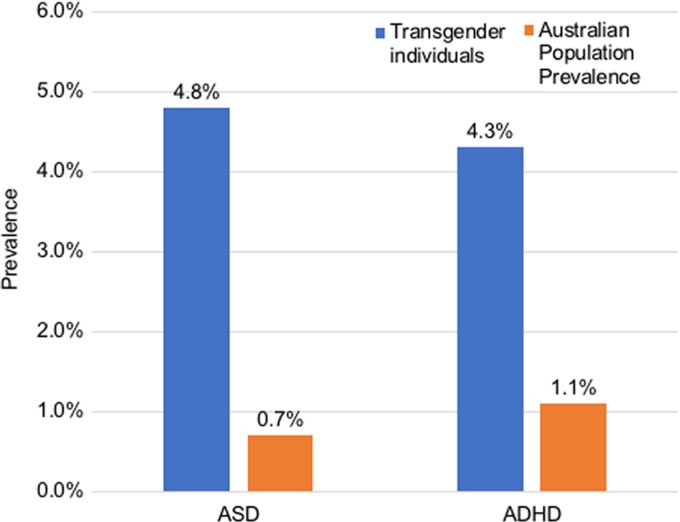
Prevalence of ASD and ADHD in our cohort and the Australian adult population prevalence.^30,31^ ADHD, attention-deficit/hyperactivity disorder; ASD, autism spectrum disorder.

Around 88.3% had been assessed by a psychiatrist or psychologist experienced in gender dysphoria before commencement of gender-affirming hormone therapy. The prevalence of psychiatric conditions was high and the most commonly diagnosed conditions are outlined in Table 3. Of note, depression was prevalent in 55.7%, anxiety in 40.4%, ASD in 4.8% and ADHD in 4.3%, all higher than age-matched Australian population means (Fig. 2).^30,31^

**Table 3.** Prevalence of Psychiatric Conditions

	Australian population prevalence^a^ %	All *N*=540
Major depression	7.9%^b62^	301 (55.7%)
Anxiety	16.3%^b62^	218 (40.4%)
Bipolar disorder	1.8–3.6%^67^	18 (3.3%)
Post-traumatic stress disorder	6.4%^67^	24 (4.4%)
Obsessive compulsive disorder	1.9%^67^	11 (2.0%)
Borderline personality disorder	2.7%–6%^68^	35 (6.5%)
Other personality disorders	<1.7%^68^	8 (1.5%)
Eating disorders	0.8–11.1%^69^	16 (3.0%)
Autism spectrum disorder	0.7%^31^	26 (4.8%)
Attention-deficit/hyperactivity syndrome (ADHD)	1.1%^30^	23 (4.3%)

Number (proportion of the cohort) is reported.

^a^ Australian population prevalence is based on median age of 27.

^b^ Refers to prevalence rates for age group 25–34.

Five individuals had diagnoses of both ASD and ADHD. Of individuals with ADHD, there were similar numbers who were birth-assigned males (*n*=12) and birth-assigned females (*n*=11). Conversely in individuals with ASD, there was a greater proportion of birth-assigned males [*n*=17 (65%)] than birth-assigned females [*n*=9 (35%)]. However, the proportion of ASD is not significantly different between birth-assigned males (8.7%) and birth-assigned females (3.8%; Chi-squared=3.74, df=1, *p*=0.053). Thirty-two percent of individuals with ADHD were taking stimulant medication.

#### Gender-affirming hormone therapy regimens

Choice of hormone therapy varied widely. The most frequently used regimen for trans females was estradiol valerate combined with either spironolactone or cyproterone acetate and for trans males, intramuscular testosterone undecanoate injections (Table 4). Preferred agents were mirrored in individuals identifying as gender nonbinary.

**Table 4.** Gender-Affirming Hormone Therapy Regimens Prescribed

	All	Proportion
Trans female individuals	*N*=177	
Oral estradiol + antiandrogen^a^	77	43.5%
Oral estradiol	45	25.4%
Transdermal estradiol (patch/gel)	11	6.2%
Transdermal estradiol (patch/gel) + oral estradiol + antiandrogen^a^	10	5.6%
Combined oral contraceptive pill^b^	8	4.5%
Transdermal estradiol (patch/gel) + antiandrogen^a^	7	4.0%
Transdermal estradiol (patch/gel) + oral estradiol	3	1.7%
Antiandrogen	2	1.1%
Combined oral contraceptive pill^b^ + antiandrogen^a^	2	1.1%
GnRH agonist subcutaneous implant	2	1.1%
GnRH agonist subcutaneous implant + oral estradiol	1	0.6%
Other^c^	9	5.1%
Trans male individuals	*N*=218	
Testosterone undecanoate injection	160	73.4%
Testosterone enanthate injection	35	16.1%
Transdermal testosterone (solution/gel/cream)	18	8.3%
Testosterone undecanoate injection + transdermal testosterone (solution/gel/cream)	2	0.9%
Testosterone enanthate injection+ transdermal testosterone (solution/gel/cream)	1	0.5%
Testosterone enanthate injection + Testosterone ester mix injection	1	0.5%
Testosterone ester mix injection	1	0.5%
Gender nonbinary individuals	*N*=51	
Birth-assigned females		
Testosterone undecanoate injection	15	29.4%
Transdermal testosterone (solution/gel)	12	23.5%
Testosterone enanthate injection	4	7.8%
Birth-assigned males		
Oral estradiol + antiandrogen^a^	11	21.6%
Oral estradiol	4	7.8%
Topical estradiol (patch) + oral estradiol + antiandrogen^a^	1	2.0%
Antiandrogen^a^	1	2.0%
Combined oral contraceptive pill	1	2.0%
Other^d^	2	3.9%

The total number refers to only individuals who were receiving gender-affirming hormone therapy. Number (proportion) is reported.

^a^ Antiandrogen refers to cyproterone acetate, spironolactone, or bicalutamide.

^b^ Ethinyl estradiol and levonorgestrel.

^c^ Other refers to raloxifene + oral estradiol + antiandrogen (*n*=1), oral estradiol + progesterone (*n*=2), transdermal estradiol (patch) + antiandrogen + progesterone (*n*=1), oral estradiol + antiandrogen + progesterone (*n*=2), estradiol/progesterone cream + antiandrogen (*n*=1), oral estradiol + low-dose transdermal testosterone (*n*=2).

^d^ Other refers to raloxifene + antiandrogen (*n*=1), transdermal estradiol (patch) + antiandrogen + progesterone (*n*=1).

#### Gender affirmation surgery

Of 196 trans female individuals, 36 (18.4%) had undergone genital reassignment surgery (vaginoplasty and orchidectomy). Four (2.0%) had orchidectomy alone, 6 (3.1%) had breast augmentation, 5 (2.6%) had facial feminization surgery and 1 individual had a laryngeal shave procedure. Of 238 trans male individuals, 14 (5.9%) had undergone hysterectomy and 1 individual had phalloplasty. Mastectomy had been performed in 88 (40.0%) individuals. Of 99 individuals identifying as gender nonbinary, 1 individual (1.0%) had orchidectomy, 11 (12.1%) had mastectomy, and 1 (1.0%) had laryngeal shave.

Among individuals with ASD and/or ADHD, of 19 trans female individuals, 1 (5%) had vaginoplasty. Of 15 trans males, 5 had prior mastectomy (33%). No individuals who identified as gender nonbinary had any prior surgery.

## Discussion

In this large cross-sectional study of adult transgender individuals, we report a rapid rise in demand for transgender health care, with more than 10 times the number in 2016 compared with 2011. Individuals identifying as gender nonbinary comprise 18.3% of the overall transgender cohort and greater numbers of trans male individuals are seen in endocrine practice. Despite few medical morbidities, smoking, unemployment, social disadvantage, and mental illness are highly prevalent which must be considered in the development of comprehensive multidisciplinary care for transgender Australians. Notably, the prevalence of ADHD, ASD, depression, and anxiety are higher in adult transgender individuals than age-appropriate Australian population mean.

### Increasing demand

Due to fear of disclosure, differences in case definition^32^ and a lack of studies involving population-based representative samples, the prevalence of gender dysphoria is inherently challenging to determine. Estimates report 0.1–0.6% of the population identify as gender variant.^33–35^ Demand for gender clinics has risen several fold in many countries worldwide over the last decade.^4,36,37^ It is not only demand for hormonal therapy, but gender-affirming surgery has also increased three-fold between 2012–2014.^38^ Our data mirror this worldwide trend, which is likely to be a result of increased societal acceptance of gender diversity, tolerance, and visibility of transgender individuals online and in the media. The concern is that current health care services will not be able to meet continuous increases in demand. Expansion of telehealth consultations may be a cost-effective strategy to provide specialized gender outreach services (in partnership with local primary care physicians) to the 30% of individuals residing in rural and regional locations.

### Gender identity

Trans females traditionally outnumber trans males,^6,39^ however, gender identity distributions observed in this study showed a shift. The endocrine specialist cohort demonstrated a reversal of this ratio with three times as many trans males as trans females presenting for hormone therapy. This is likely related to new restrictions by the Australian Pharmaceutical Benefits Scheme introduced in early 2015, which require a specialist endocrinologist, sexual health physician, or urologist consultation to access government-subsidized testosterone therapy. It is also possible that our results may reflect changing gender proportions within the transgender community at large. Indeed, this latter argument is supported by the ongoing multinational ENIGI study recently documenting higher frequencies of trans male individuals in their combined study population^40^ and in Canadian and Dutch gender clinics.^41^

We report a high prevalence of nonbinary gender identity (30%) among our primary care population. There are no peer-reviewed publications examining the prevalence of nonbinary gender identity, however, published online surveys of transgender populations report between 5% and 13% identity as such.^42,43^ Identification as a nonbinary gender, of which there are many terms with which individuals identify (i.e., genderqueer, gender fluid, agender), may be emerging with increasing recognition of gender as a spectrum and societal acceptance in challenging conventional gender stereotypes.

### Sociodemographic characteristics

Despite relatively high levels of education, unemployment rates of 21.3% were high in this relatively young cohort, four-fold higher than the Australian general population unemployment rate of 5–6%.^44^ High rates of unemployment among transgender individuals (33–35%) have also been reported in the United States of America and Spain.^45,46^ There are a range of potential contributing factors to unemployment, including fear of disclosure, employer discrimination, conflicting gender codes or names on qualifications or references, and mental health conditions. Of concern, young transgender individuals reporting difficulties obtaining employment had higher rates of suicide attempts and mental health conditions than those who did not.^47^ Smoking rates in the transgender community are high, but similar to rates among Australians with depression and anxiety, which may reflect smoking being a means to relieve psychiatric symptoms. Smoking and being overweight are both cardiovascular risk factors affecting this relatively young population, and given the uncertain long-term cardiovascular effects of testosterone or estradiol therapy, proactive cardiovascular risk reduction should be considered in all transgender individuals.

### Clinical characteristics, ASD, and ADHD

We report a high prevalence of ADHD (4.1%) in our cohort, which is higher than adult Australian population prevalence of 1.1–2.7%.^30^ We interestingly observed a similar male to female ratio in those with ADHD, which is in contrast to the male predominance observed in the general population.^48^ However, it has been suggested that underdiagnoses may occur among females in the community,^48^ and symptoms, which are typically more commonly exhibited by women, such as inattention, may not be recognized as symptoms of ADHD.^49^ There has only been one other small study suggesting a higher prevalence of ADHD in adult transgender individuals (*n*=54).^19^ Notably, this was a convenience sample from a paid online survey, which has multiple limitations, including a bias toward young internet users, and is unlikely to be representative of population-based samples or transgender cohorts.^19^ Potentially, misdiagnoses of ADHD may be a contributing factor. Both ASD and ADHD can severely compromise health and wellbeing, particularly if left undiagnosed.^30,50^ Symptoms such as attention difficulties, deficits in communication and social skills, obsessional interests, and stereotyped behavior can significantly impact assessment of gender dysphoria, understanding of health information, and engagement in clinical care.^51^ These factors may potentially explain fewer individuals with ASD or ADHD undergoing gender-affirming surgery than transgender individuals in our study. Gender nonconforming youth often present with externalizing behaviors,^52^ and symptoms such as attention deficit, impulsivity, and hyperactivity may be explained in part by ASD rather than ADHD, although distinguishing the two diagnoses is usually achievable.^53^ While contentious and lacking supportive data, it has been suggested that endocrine disruptors such as prenatal exposure to phthalates^54^ or antidepressants^55^ may be an explanation for the increase of ADHD and ASD and relationship with gender variance. There is evidence that phthalates may play a role in reproductive development *in utero* and sex steroid hormone levels.^56,57^ Sex hormones may also play a role in the development of these conditions. Familial and twin studies have shown that approximately 50–72% of contributing genetic factors overlap in ADHD and ASD.^58^ There may potentially be shared genetic or epigenetic underpinnings or neurodevelopmental links among gender identity, ADHD, and ASD.^59^ Such hypotheses and observations will hopefully provide impetus to further study the links among ADHD, ASD, and gender.

The prevalence of ASD of 5% in our adult cohort is also significantly higher than Australian adult population prevalence rates of <1%.^60^ Three published cross-sectional analyses of clinical chart data have described similar rates of prior diagnoses of ASD ranging from 6% in adults and 7–13.3% in children and adolescents with gender dysphoria.^12,13,16^ Many more studies have used surrogate autism screening tools and reported scores suggestive of ASD, however, it is possible that high levels of social anxiety related to minority stress or potential peer or family rejection may lead to falsely positive screening tests.^61^ Prospective controlled studies with rigorous diagnostic assessments for ASD, ADHD, and gender dysphoria are required.

Not surprisingly, we report an extremely high prevalence of diagnosed depression and anxiety, which is 10-fold higher than the general population^62^ and is comparable with previous studies among transgender populations.^6,8,46,63^ There are greater risks of depression, anxiety, and suicide reported among individuals with ADHD and ASD.^64,65^ Although there are many contributing factors to this, including discrimination and difficulties accessing gender-affirming treatments, our results exemplify the need for multidisciplinary coordinated care^66^ and mental health support for transgender individuals, including availability of services to treat, monitor, and effectively support individuals with ASD and ADHD. As both ASD and ADHD are complex conditions with variable functional challenges, provision of information and gender-affirming care needs to be tailored to different learning styles specific to the individual. Clinicians may need to employ a range of different tools and approaches to account for factors such as inattention, lack of organization, and communication difficulties. Additionally, psychosocial interventions to develop interpersonal skills, increase self-esteem, and improve social and peer support may benefit individuals who struggle with social aspects of gender transition.

There were a number of key limitations to this cross-sectional retrospective audit. While we attempted to capture primary and specialist care clinics in the state of Victoria, we are uncertain of referral patterns in other states and territories of Australia, however, given the worldwide trends reported, it is likely similar rises in demand for transgender health are being seen. In addition to limitations inherent in the study design, including lack of a control group and missing data, we collected diagnoses listed in medical records, which while entered by medical practitioners during initial consultations, are largely based on self-reported clinical history and we did not have details regarding the specific diagnostic process leading to the diagnosis of ADHD or ASD. We also did not have temporal data on the effect of gender-affirming hormone therapy on mental health conditions such as depression. We also did not have data on suicidality or contributing factors to depression or anxiety. Nonetheless, this is a large real-life clinical cohort and our findings warrant further confirmation in prospective studies. Until further data are available, decreasing mental health burden and improving quality of life should be made a priority. Comprehensive multidisciplinary gender services may be best placed to meet the complex mental health needs of this socially disadvantaged group.

## Conclusion

There is a rising demand for transgender health services, and we observe an increased prevalence of depression, anxiety, ADHD, and ASD higher than the general population. A coordinated multidisciplinary approach to transgender health care, including psychosocial interventions to support mental health and neurobehavioral conditions in adults in parallel to gender-affirming treatments are essential to meet the needs of this socially disadvantaged cohort. There are many future research priorities, including studies to assess and understand the links between neurobehavioral conditions and gender dysphoria, clinical trials to provide evidence-based treatment pathways and studies to evaluate optimal models of health service provision to improve quality of life and minimize mental health burden. Until further evidence is available, provision of health services need to be tailored to the specific health needs of transgender individuals with a focus on continual quality improvement as new knowledge and data arise.

## Abbreviations Used

ADHD: attention-deficit/hyperactivity disorder
ASD: autism spectrum disorder
BMI: body mass index
